# Domestic

**Published:** 1842-01-08

**Authors:** 


### DOMESTIC.

*Extraction of the Primary Teeth—Cutting the Gums.*—It is sometimes adviseable to recall attention to well known surgical or physiological facts, which are apt to escape the attention of medical men, because they are usually considered as appertaining more particularly to the duties of specialists, though, if fairly considered, they would be found at least equally important to the general practitioner (we use the words in the American sense) when placed at a distance from cities where the division of professional labour is carried to the greatest extent. For this reason, we take from the Baltimore Guardian of Health, for December, the following short extracts from a paper by Enoch Noyes, M. D., Dental surgeon.

"The opinion that the preservation of the teeth of the first dentition, is unimportant, inasmuch as they are soon removed by the operation of nature, and are then succeeded by those of the second—a larger, stronger, and withal more numerous set—is not only erroneous, but is fraught with danger to the health of both.—"Dr. Harris, in treating upon the importance of the preservation of the first teeth, to the health and durability of the second, says: 'It is very important, that the deciduous teeth should be preserved until they are removed by the absorption of their fangs; because, in their health is involved the durability and health of the permanent teeth.'—"When the first teeth continue healthy until, by the destruction of their roots, they are removed to give place to the second, these latter are generally well arranged, of a hard and firm texture, and, in consequence, less liable to disease than when developed in the midst of unhealthy parts, as they usually are when the former are allowed to decay, for it is to this, that the morbid affections which are so often met with during early childhood, in their sockets and surrounding gums, are in the majority of cases attributable. The teeth of the second set are formed behind and beneath those of the first, and any unhealthy condition of these latter or their contiguous parts, proportionate to its nature and extent, disturbs the process of the formation of the former. The earthy material that enters into their composition, is caused to be by it less abundant and inferior in quality."

The early and careless extraction of the first set of teeth by empirical dentists, and by medical men who regard such subjects as unworthy of close attention, has been not only the ruin of the appearance, but a serious injury to the health of many a child under our own observation. The lancing of gums too early and too deeply, before the crowns of the teeth are properly solidified, is another and very gross error of practice. Almost the only complaint of childhood, in which a neglect of these principles is warrantable, is the *cancrum oris,* or sore mouth disease of children, in which it is essential that our local remedies—the saturated solution of sulphate of copper, for instance—should be carried to the very bottom of the socket. Life is placed in immediate and imminent jeopardy in every case of this disease, and, unfortunately, the teeth must be sacrificed to the requisite extent without reserve.

